# Discovery of novel naphthalene-based diarylamides as pan-Raf kinase inhibitors with promising anti-melanoma activity: rational design, synthesis, in vitro and in silico screening

**DOI:** 10.1007/s12272-025-01533-5

**Published:** 2025-02-08

**Authors:** Ahmed Elkamhawy, Usama M. Ammar, Minkyoung Kim, Anam Rana Gul, Tae Jung Park, Kyeong Lee

**Affiliations:** 1 Department of Chemistry, School of Sciences and Humanities, Nazarbayev University, Kabanbay Batyr 53, Astana, 010000 Kazakhstan; 2 School of Applied Sciences, Edinburgh Napier University, Sighthill Campus, 9 Sighthill Court, Edinburgh, EH11 4BN UK; 3 BK21 FOUR Team and Integrated Research Institute for Drug Development, College of Pharmacy, Dongguk University-Seoul, Goyang, 10326 Republic of Korea; 4 Department of Chemistry, Research Institute of Chem-Bio Diagnostic Technology, Chung-Ang University, 84 Heukseok-Ro, Dongjak-Gu, Seoul, 06974 Republic of Korea

**Keywords:** Pan-Raf kinase inhibitors, Drug design, Naphthalene-based derivatives, Difluoromethoxy group, Anticancer drug, Melanoma

## Abstract

Raf kinase enzymes are often dysregulated in melanoma. While sorafenib demonstrates strong activity against wild-type B-Raf, it fails to effectively inhibit the mutated form of B-Raf. In this study, sorafenib served as a lead compound for the development of new derivatives designed to enhance inhibitory activity across multiple Raf isoforms (pan-Raf inhibitors). Novel naphthalene-based diarylamide derivatives were subsequently designed, synthesized, and evaluated for their biological activity against various Raf kinase isoforms and the melanoma A375 cell line. Among these, compound **9a**, containing a difluoromethoxy group, demonstrated strong inhibitory activity across B-Raf^WT^, B-Raf^V600E^, and c-Raf. Additionally, it induced G2/M phase arrest and triggered dose-dependent apoptosis, effectively suppressing both cell proliferation and survival. Compound **9a** also exhibited high selectivity for Raf isoforms with minimal off-target effects, underscoring its specificity and therapeutic potential for Raf-driven malignancies.

**Supplementary Information:**

The online version contains supplementary material available at 10.1007/s12272-025-01533-5.

## Introduction

Cancer is a malignant condition characterized by uncontrolled cell growth and proliferation, resulting from mutations or overexpression in critical biological targets within the human body (Ammar et al. 2018; Abdel-Maksoud et al. 2021). MAPK signalling cascade is one of signalling pathways within the human cell, from cell membrane to nucleus (Wellbrock et al. 2004; Buchstaller et al. 2011). It has been reported that MAPK signalling cascade is significantly related to the progression of a variety of human cancers (Ammar et al. 2020; Zhao et al. 2022). MAPK signalling pathway consists of Ras/Raf/MEK/ERK signal transduction cascade that plays an important role in a number of cellular activities such as cell growth, cell proliferation, cell survival, and other biological aspects that contribute to cellular behaviour (Pritchard et al. 1995; Weber et al. 2000; Minamoto et al. 2000; Yuen et al. 2002; El-Damasy et al. 2020). The vital roles of these biological cellular components make MAPK signalling pathway an attractive biological target in treating human cancers (Weber et al. 2000; El-Damasy et al. 2020).

Raf protein kinases (A-Raf, B-Raf, and c-Raf) interact significantly with Ras to activate MAPK signalling pathway (Weber et al. 2000; Yuen et al. 2002; Buchstaller et al. 2011). In addition, it has been reported that B-Raf isoform binds and activates c-Raf in a RAS-dependant manner that reflects the key role of B-Raf within the MAPK signalling pathway (Garnett et al. 2005; Rushworth et al. 2006; Buchstaller et al. 2011). Following Raf activation in response to external stimuli (Chiloeches and Marais 2002), the signalling cascade is triggered, and downstream regulators (MEK and ERK) are sequentially phosphorylated and activated to regulate the cellular biological activities (Kolch 2000; Li et al. 2009; Zhan et al. 2012). Therefore, the inhibition of MAPK signalling pathway at the level of Raf protein kinases is expected to be an effective and promising therapeutic strategy to treat human cancers driven through this signalling cascade (El-Damasy et al. 2020). It has been reported that B-Raf kinase mutation (V600E) in skin is a critical step in prompting melanoma disease (Pollock et al. 2003). In the V600E single-point mutation, a glutamic acid residue replaces the valine residue at position 600. The replacement of a small, non-polar valine residue with a larger, negatively charged glutamic acid causes a conformational change in the ATP active site of the kinase domain, mimicking the phosphorylation of the activation segment. This mutation causes a conformational shift from the inactive state (wild-type B-RAF) to the active state (V600E BRAF). Consequently, it results in the constitutive activation of B-RAF kinase activity, bypassing upstream signals and leading to the continuous activation of downstream proteins (MEK/ERK). V600E mutation in the kinase domain of B-Raf protein accounted for 66% of melanoma, 40–70% of papillary thyroid carcinoma, 12% of colon carcinomas, and 14% of liver cancers (Yuen et al. 2002, ET et al. 2003, El-Damasy et al. 2020). The significant effects of Raf protein kinases on cellular activities, together with high prevalence of mutation in melanoma, make Raf an attractive biological target for treatment of human melanoma disease (Ammar et al. 2018; El-Damasy et al. 2020).

**Fig. 1:**
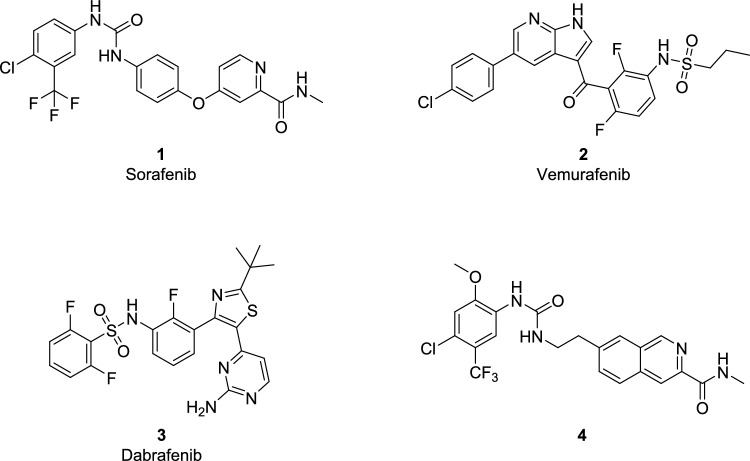
The reported Raf inhibitors

Targeting the oncogenic protein kinases has been reported to be an effective therapeutic strategy to treat cancer diseases (Bhullar et al. 2018; Lee et al. 2021). By early 2024, the Food and Drug Administration (FDA) has approved a total of fifty-nine small molecule kinase inhibitors (SMKIs) for treating oncology-related conditions (Roskoski 2024). It was reported that the design and development of new drugs targeting Raf kinases showed paradoxical activation of MAPK signalling cascade following Raf inhibition (Zhao et al. 2022). In addition, the first reported pan-Raf inhibitor BAY43-996 (Sorafenib **1**), showed weak efficacy in patients with B-Raf mutation (V600E)-based melanoma (Smith et al. 2001; Khire et al. 2004; Ramurthy et al. 2008). It was suggested that sorafenib showed an additional mechanism of action in targeting melanoma through inhibition of VEGFR kinase domain (Wilhelm et al. 2004; Eisen et al. 2006; Ramurthy et al. 2008; Zhan et al. 2012; El-Damasy et al. 2020). Moreover, selective B-Raf (V600E) inhibitors (Vemurafenib **2** and Dabrafenib **3**, Fig. 1) showed significant clinical response in metastatic melanoma with oncogenic B-Raf mutation (V600E) (Ali et al. 2022). However, these FDA drugs (**2** and **3**) failed to afford sustained tumour remission in cancers with wild B-Raf protein kinase (WT) (Zhao et al. 2022). In addition, these first-generation Raf inhibitors showed a number of adverse events such as the development of cutaneous cell tumour, keratoacanthomas and acquired resistance (Shaw et al. 2014). Acquired resistance significantly reduces the effectiveness of these inhibitors, restricting their therapeutic benefits to a limited period of six to nine months. (Rajakulendran et al. 2009; Shaw et al. 2014; Agianian and Gavathiotis 2018). As a result, the development of next-generation small molecule kinase inhibitors (SMKIs), through targeting pan-Raf protein kinases (targeting all Raf isoforms), has attracted great interests from both pharmaceutical industry and academia (Yang et al. 2015; Wang et al. 2017).

**Fig. 2:**
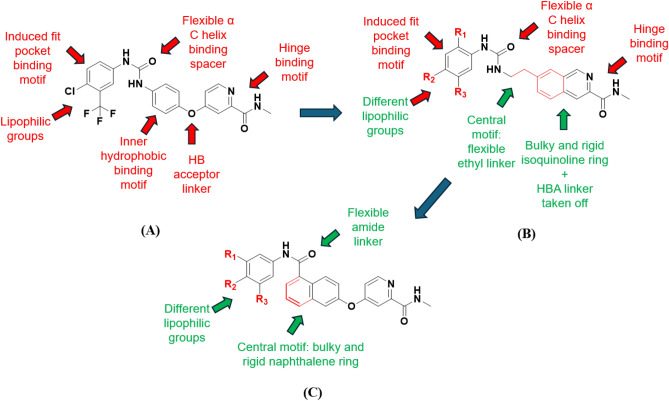
The rational design of the development of a new series of sorafenib-related derivatives. **A** the structural feature of sorafenib as a lead compound in this current study; **B** the general structure of developed compounds by Buchstaller group in 2011; **C** the proposed structural feature of the newly designed compounds by our research group. Where the fused six-membered ring, naphthalene, was incorporated as a central core scaffold in the designed compounds; HBD/HBA urea group was replaced with amide group with different chemical geometry; the distal phenyl ring was decorated with different non-polar groups; the terminal *N*-methyl amide group was retained in the newly designed series

This inspired us to develop novel pan-Raf inhibitors with enhanced activity across multiple Raf isoforms, including mutated B-Raf and c-Raf. Sorafenib was selected as a lead compound for molecular development to elucidate the Structure-Activity Relationship (SAR) of newly designed sorafenib-based derivatives. Strategic structural modifications were implemented to optimize its inhibitory activity across the Raf kinase isoforms. In 2011, Buchstaller research group implemented structural modifications to sorafenib to improve its activity against B-Raf kinase (Fig. 1; compound **4**, and Fig. 2B) (Buchstaller et al. 2011). They applied structure rigidification to the distal pyridine ring of sorafenib and replaced it with isoquinoline ring. The central phenyl ring was replaced by a flexible ethyl linker between the distal isoquinoline ring and the urea motif. In addition, they have decorated the terminal phenyl ring with different lipophilic substituents to study the effect of hydrophobic interaction with the target protein. However, they retained the *N*-methyl amide group (hinge binding motif) and urea-based linker (H-bonding-forming group) together with the extended conformation. This approach identified compound **4** as the most potent in their series, with an IC_50_ value of 80 nM against the Raf kinase enzyme.

Accordingly, our rational design was inspired by the sequential drug development strategies applied to sorafenib and the drug-like candidate developed by the Buchstaller group (Fig. 2 and Table 1). Our newly designed compounds retained the stretched conformation of sorafenib and conserved the characteristic terminal *N*-methyl amide group. We replaced the central phenyl ring with a more rigid and bulkier naphthalene ring. This central aromatic structure has been established as a key scaffold in several small-molecule kinase inhibitors (Harmange et al. 2008). Naphthalene core may also afford π staking interaction with the key amino acid residue within the inner hydrophobic pocket (Phe545). The urea-based linker was replaced with an amide group to investigate the potential of this new chemical geometry within the HBD/HBA segment to form hydrogen bonds with the key amino acid residues in the α-helix (Glu501 and Asp594). Additionally, we expanded our study to investigate the SAR by substituting the terminal phenyl ring to target the induced-fit pocket. Various substituents were introduced to the terminal phenyl ring to evaluate their impact on interactions within the hydrophobic pocket.

**Table 1:** Chemical structures and the isolated yields of compounds **9a**–**i**


Compound	R_1_	R_2_	R_3_	Yield%
**9a**		H	H	80.5
**9b**	H		H	85.6
**9c**	H	I	H	90.1
**9d**	F	OMe	H	92.3
**9e**	F		H	95.6
**9f**	H	F	H	76.3
**9g**	Me	Me	H	87.8
**9h**	Me	H	Br	94.3
**9i**	Cl	OMe	H	82.1

## Materials and methods

### Chemistry

The general protocols used for chemical synthesis, structure elucidation, and purity assessment of the synthesized compounds followed previously reported methods (Al-Sanea et al. 2015; Park et al. 2017; Elkamhawy et al. 2020; Elsherbeny et al. 2021). In brief, all solvents and reagents were used without any additional purification. The ^1^H NMR data were obtained using a Varian 400 MHz spectrometer (Varian Medical Systems, Inc., Palo Alto, CA, USA) and measured in parts per million (ppm) for chemical shifts and in Hz for coupling constants. High-resolution electrospray ionization mass spectrometry (HR-ESIMS) data were analyzed using a JMS-700 mass spectrometer or HR-ESIMS data obtained via a G2 QTOF mass spectrometer (Waters Corporation, Milford, MA, USA). Reaction progress was monitored using TLC on silica plates with a thickness of 0.25 mm from E. Merck and silica gel 60 F_254_. The purity of the final compounds was confirmed using reverse-phase high-performance liquid chromatography (Biotage, Uppsala, Sweden) equipped with a UV detector set at 254 nm. The mobile phases used were H_2_O containing 0.05% trifluoroacetic acid and CH_3_CN. HPLC used an YMC Hydrosphere C_18_ (HS-302) column with a diameter of 4.6 mm and a length of 150 mm, with a flow rate of 1.0 mL/min.

### Synthesis of the intermediate naphthamides 7a–i

The intermediate compounds **7a**–**i** were synthesized as reported (Elkamhawy et al. 2024).

### Synthesis of the final compounds 9a–i

In a round-bottom flask containing 5 mL of dimethyl sulfoxide (DMSO), an amount of 0.13 g (0.9 mmol) of 4-chloro-*N*-methylpicolinamide (**8**) and cesium carbonate (Cs_2_CO_3_, 0.58 g, 1.8 mmol, 3 equiv) were dissolved at room temperature. After stirring for 15 min, the appropriate naphthamide intermediate (**7a**–**i,** 1.13 mmol, 0.6 equiv) was added. The reaction mixture was then stirred for 12 h at 110 °C. After completion of the reaction, the mixture was allowed to cool to rt. The excess solvent was then evaporated, and the residue was partitioned between water and EtOAc. Anhydrous magnesium sulfate was added to the wet organic layer and the resulting solid was removed by filtration. The filtrate was then evaporated under reduced pressure and the solid was washed with *n*-hexane and purified using flash column chromatography (EtOAc/*n*-hex: 1/1) to obtain the final product.

### 4-((5-((3-(Difluoromethoxy)phenyl)carbamoyl)naphthalen-2-yl)oxy)-N-methylpicolinamide (9a)

Off-white solid. Yield: 80.5%. Mp: 150.3–151.2 °C. ^1^H NMR (400 MHz, DMSO-*d*_*6*_) *δ* 10.83 (s, 1H), 8.80 (s, 1H), 8.57 (d, *J* = 5.7 Hz, 1H), 8.32 (d, *J* = 9.1 Hz, 1H), 8.12 (d, *J* = 8.6 Hz, 1H), 7.90 (s, 1H), 7.83–7.76 (m, 2H), 7.72–7.59 (m, 2H), 7.54–7.39 (m, 3H), 7.31–7.22 (m, 1H), 6.95 (d, *J* = 7.3 Hz, 1H), 2.78 (d, *J* = 4.7 Hz, 3H). ^13^C NMR (100 MHz, DMSO-*d*_*6*_) *δ* 167.71, 165.80, 164.17, 153.05, 151.61, 151.02, 141.12, 135.03, 134.73, 130.59, 130.42, 128.60, 127.89, 126.65, 125.95, 122.05, 119.41, 118.39, 116.91, 116.85, 115.06, 114.24, 110.78, 109.76, 26.44. HRMS (ESI) *m/z* calc. for C_25_H_20_F_2_N_3_O_4_ [M + H]^+^ 464.1422, found 464.1455. Purity: 97.32% (as determined by RP-HPLC, R_t_ = 17.5 min).

### 4-((5-((4-Isopropylphenyl)carbamoyl)naphthalen-2-yl)oxy)-N-methylpicolinamide (9b)

Off-white solid. Yield: 85.6%. Mp: 160.3–161.7 °C. ^1^H NMR (400 MHz, DMSO-*d*_*6*_) *δ* 10.55 (s, 1H), 8.84–8.75 (m, 1H), 8.56 (d, *J* = 5.8 Hz, 1H), 8.31 (d, *J* = 9.0 Hz, 1H), 8.09 (d, *J* = 8.3 Hz, 1H), 7.89 (d, *J* = 2.3 Hz, 1H), 7.80–7.65 (m, 4H), 7.52–7.42 (m, 2H), 7.33–7.17 (m, 3H), 2.78 (d, *J* = 4.9 Hz, 3H), 1.21 (d, *J* = 6.9 Hz, 6H). ^13^C NMR (100 MHz, DMSO-*d*_*6*_) *δ* 167.28, 165.83, 164.18, 153.05, 151.68, 151.02, 144.38, 137.41, 135.55, 134.72, 130.09, 128.71, 128.00, 126.86, 126.68, 125.74, 121.90, 120.47, 118.35, 115.04, 109.76, 33.39, 26.44, 24.41. HRMS (ESI) *m/z* calc. for C_27_H_26_N_3_O_3_ [M + H]^+^ 440.1974, found 440.1965. Purity: 98.37% (as determined by RP-HPLC, R_t_ = 20.0 min).

### 4-((5-((4-Iodophenyl)carbamoyl)naphthalen-2-yl)oxy)-N-methylpicolinamide (9c)

Yellow solid. Yield: 90.1%. Mp: 134.0–134.5 °C. ^1^H NMR (400 MHz, DMSO-*d*_*6*_) *δ* 10.74 (s, 1H), 8.79 (s, 1H), 8.56 (d, *J* = 5.3 Hz, 1H), 8.30 (d, *J* = 9.2 Hz, 1H), 8.11 (d, *J* = 8.2 Hz, 1H), 7.90 (d, *J* = 2.3 Hz, 1H), 7.83–7.62 (m, 5H), 7.52–7.42 (m, 2H), 7.30–7.23 (m, 1H), 2.78 (d, *J* = 4.9 Hz, 3H). ^13^C NMR (100 MHz, DMSO-*d*_*6*_) *δ* 167.57, 165.80, 164.17, 153.05, 151.73, 151.03, 150.45, 139.48, 137.85, 135.10, 134.72, 130.37, 128.61, 127.89, 126.65, 125.93, 122.52, 122.23, 122.02, 118.38, 115.06, 109.76, 87.83, 26.45. HRMS (ESI) *m/z* calc. for C_24_H_18_IN_3_O_3_ [M + H]^+^ 524.0393, found 524.0475. Purity: 94.49% (as determined by RP-HPLC, R_t_ = 19.0 min).

### 4-((5-((3-Fluoro-4-methoxyphenyl)carbamoyl)naphthalen-2-yl)oxy)-N-methylpicolinamide (9d)

Off-white solid. Yield: 92.3%. Mp: 198.3–199.2 °C. ^1^H NMR (400 MHz, DMSO-*d*_*6*_) *δ* 10.65 (s, 1H), 8.80 (d, *J* = 5.0 Hz, 1H), 8.56 (d, *J* = 5.6 Hz, 1H), 8.32 (d, *J* = 9.0 Hz, 1H), 8.10 (d, *J* = 8.6 Hz, 1H), 7.92–7.86 (m, 1H), 7.83–7.74 (m, 2H), 7.71–7.64 (m, 1H), 7.54–7.42 (m, 3H), 7.30–7.14 (m, 2H), 3.84 (s, 3H), 2.78 (d,* J* = 4.9 Hz, 3H). ^13^C NMR (100 MHz, DMSO-*d*_*6*_) *δ* 167.25, 165.80, 164.17, 153.04, 151.72, 151.01, 135.16, 134.73, 130.29, 128.69, 127.93, 126.64, 125.85, 121.97, 118.36, 116.35, 115.04, 114.56, 109.76, 108.65, 56.69, 26.44. HRMS (ESI) *m/z* calc. for C_25_H_21_FN_3_O_4_ [M + H]^+^ 446.1516, found 446.1512. Purity: 98.02% (as determined by RP-HPLC, R_t_ = 15.2 min).

### 4-((5-((3-Fluoro-4-morpholinophenyl)carbamoyl)naphthalen-2-yl)oxy)-N-methylpicolinamide (9e)

Off-white solid. Yield: 95.6%. Mp: 177.5–179.2 °C. ^1^H NMR (400 MHz, DMSO-*d*_*6*_) *δ* 10.67 (s, 1H), 8.80 (d, *J* = 4.9 Hz, 1H), 8.56 (d, *J* = 5.6 Hz, 1H), 8.31 (d, *J* = 9.3 Hz, 1H), 8.10 (d, *J* = 8.0 Hz, 1H), 7.89 (d, *J* = 2.5 Hz, 1H), 7.80–7.63 (m, 3H), 7.53–7.41 (m, 3H), 7.27 (dd, *J* = 5.5, 2.7 Hz, 1H), 7.07 (t, *J* = 9.4 Hz, 1H), 3.79–3.70 (m, 3H), 3.03–2.95 (m, 3H), 2.78 (d, *J* = 4.9 Hz, 2H), 2.54 (s, 3H). ^13^C NMR (100 MHz, DMSO-*d*_*6*_) *δ* 167.27, 165.81, 164.17, 156.07, 153.65, 153.04, 151.71, 151.03, 136.11, 135.17, 134.67, 130.30, 128.67, 127.93, 126.66, 125.86, 121.98, 119.59, 118.37, 116.42, 115.05, 109.75, 108.72, 108.46, 66.64, 51.24, 51.21, 26.45. HRMS (ESI) *m/z* calc. for C_28_H_26_FN_4_O_4_ [M + H]^+^ 501.1938, found 501.1931. Purity: 99.01% (as determined by RP-HPLC, R_t_ = 14.6 min).

### 4-((5-((4-Fluorophenyl)carbamoyl)naphthalen-2-yl)oxy)-N-methylpicolinamide (9f)

White solid. Yield: 76.3%. Mp: 157.3–157.8 °C. ^1^H NMR (400 MHz, DMSO-*d*_*6*_) *δ* 10.69 (s, 1H), 8.85–8.75 (m, 1H), 8.56 (d, *J* = 5.5 Hz, 1H), 8.32 (d, *J* = 9.2 Hz, 1H), 8.10 (d, *J* = 8.2 Hz, 1H), 7.92–7.75 (m, 3H), 7.72–7.63 (m, 1H), 7.47 (dd, *J* = 19.9, 7.0 Hz, 2H), 7.31–7.17 (m, 2H), 2.78 (d, *J* = 4.8 Hz, 3H). ^13^C NMR (100 MHz, DMSO-*d*_*6*_) *δ* 167.36, 165.81, 164.17, 153.04, 151.70, 151.02, 136.01, 135.24, 134.73, 130.26, 128.67, 127.94, 126.67, 125.86, 122.18, 122.10, 121.98, 118.39, 115.87, 115.65, 115.05, 109.74, 26.45. HRMS (ESI) *m/z* calc. for C_24_H_19_FN_3_O_3_ [M + H]^+^ 416.4140, found 416.1406. Purity: 98.07% (as determined by RP-HPLC, R_t_ = 15.4 min).

### 4-((5-((3,4-Dimethylphenyl)carbamoyl)naphthalen-2-yl)oxy)-N-methylpicolinamide (9 g)

Off-white solid. Yield: 87.8%. Mp: 112.2–113.5 °C. ^1^H NMR (400 MHz, DMSO-*d*_*6*_) *δ* 10.47 (s, 1H), 8.83–8.76 (m, 1H), 8.56 (d, *J* = 5.8 Hz, 1H), 8.31 (d, *J* = 9.1 Hz, 1H), 8.09 (d, *J* = 8.6 Hz, 1H), 7.89 (d, *J* = 2.2 Hz, 1H), 7.79–7.85 (m, 1H), 7.70–7.57 (m, 2H), 7.56–7.41 (m, 3H), 7.30–7.23 (m, 1H), 7.12 (d, *J* = 8.2 Hz, 1H), 2.78 (d, *J* = 4.9 Hz, 3H), 2.22 (d, *J* = 9.9 Hz, 6H). ^13^C NMR (100 MHz, DMSO-*d*_*6*_) *δ* 167.23, 165.83, 164.17, 153.03, 151.65, 151.03, 137.37, 136.74, 135.61, 134.71, 131.99, 129.99, 128.70, 128.00, 126.69, 125.70, 121.89, 121.59, 118.36, 117.93, 115.03, 109.73, 26.45, 20.10, 19.29. HRMS (ESI) *m/z* calc. for C_26_H_24_N_3_O_3_ [M + H]^+^ 426.1818, found 426.1820. Purity: 99.52% (as determined by RP-HPLC, R_t_ = 17.4 min).

### 4-((5-((3-Bromo-5-methylphenyl)carbamoyl)naphthalen-2-yl)oxy)-N-methylpicolinamide (9 h)

Yellow solid. Yield: 94.3%. Mp: 132.9–133.7 °C. ^1^H NMR (400 MHz, DMSO-*d*_*6*_) *δ* 10.72 (s, 1H), 8.83–8.77 (m, 1H), 8.57 (d, *J* = 5.6 Hz, 1H), 8.31 (d, *J* = 9.3 Hz, 1H), 8.11 (d, *J* = 8.3 Hz, 1H), 7.97–7.88 (m, 2H), 7.79 (d, *J* = 6.7 Hz, 1H), 7.71–7.64 (m, 1H), 7.59 (s, 1H), 7.53–7.43 (m, 2H), 7.30–7.25 (m, 1H), 7.18 (s, 1H), 2.78 (d, *J* = 4.8 Hz, 3H), 2.32 (s, 3H). ^13^C NMR (100 MHz, DMSO-*d*_*6*_) *δ* 167.67, 165.79, 164.16, 153.04, 151.74, 151.03, 141.03, 140.94, 134.99, 134.72, 130.43, 128.62, 127.88, 127.37, 126.65, 125.94, 122.04, 121.78, 119.91, 119.68, 118.39, 115.06, 109.75, 26.45, 21.32. HRMS (ESI) *m/z* calc. for C_25_H_21_BrN_3_O_3_ [M + H]^+^ 490.0766, found 490.0769. Purity: 97.34% (as determined by RP-HPLC, R_t_ = 19.8 min).

### 4-((5-((3-Chloro-4-methoxyphenyl)carbamoyl)naphthalen-2-yl)oxy)-N-methylpicolinamide (9i)

Yellow solid. Yield: 82.1%. Mp: 202.0–202.4 °C. ^1^H NMR (400 MHz, DMSO-*d*_*6*_) *δ* 10.61 (s, 1H), 8.83–8.73 (m, 1H), 8.55 (d, *J* = 5.6 Hz, 1H), 8.31 (d, *J* = 8.9 Hz, 1H), 8.08 (d, *J* = 7.9 Hz, 1H), 7.98 (d, *J* = 2.8 Hz, 1H), 7.88 (d, *J* = 2.4 Hz, 1H), 7.77 (d, *J* = 6.8 Hz, 1H), 7.69–7.61 (m, 2H), 7.51–7.41 (m, 2H), 7.28–7.21 (m, 1H), 7.17 (d, *J* = 9.0 Hz, 1H), 3.84 (s, 3H), 2.77 (d, *J* = 4.9 Hz, 3H). ^13^C NMR (100 MHz, DMSO-*d*_*6*_) *δ* 167.26, 165.81, 164.17, 153.03, 151.71, 151.35, 151.03, 135.12, 134.73, 133.32, 130.31, 128.71, 127.93, 126.66, 125.88, 121.98, 121.06, 120.26, 118.37, 115.05, 113.38, 109.75, 56.68, 26.45. HRMS (ESI) *m/z* calc. for C_25_H_21_ClN_3_O_4_ [M + H]^+^ 462.1221, found 462.1219. Purity: 98.49% (as determined by RP-HPLC, R_t_ = 15.4 min).

### Biological evaluation

#### In vitro biochemical kinase assay

The in vitro kinase inhibitory assays (both one-point and ten-point assays) were performed at Reaction Biology Co. (Malvern, PA, USA). Kinase HotSpot service was used for screening the tested derivatives. The experiment protocol is previously described in detail (Elkamhawy et al. 2016).

#### In vitro cytotoxic activity

Melanoma skin cancer cell line (A375) of American Type Culture Collection (ATCC) was obtained from Korean Cell Line Bank (KCLB). The A375 cells were cultured in Dulbecco’s modified eagle’s medium (DMEM) (GenDepot) supplemented with 1% penicillin–streptomycin and 10% fetal bovine serum (FBS). The cells were maintained at 37 °C in a 5% CO_2_ with a 95% humid atmosphere. MTT assay, analysis of the cell cycle distribution, apoptosis analysis, and statistical analysis were carried out following known standard protocols adopted by our team earlier (Son et al. 2023).

#### In silico molecular simulation

The molecular docking simulation of the designed derivatives (**9a**–**i**) and the standard compounds (**1** and **4**) was performed using Molecular Graphics Laboratory (MGL) Tools software suite 1.5.7 (Sanner lab, Centre for Computational Structural Biology, Scripps Research Institute) (Elkamhawy et al. 2024). The molecular docking protocol was conducted through the following steps:

### Ligand preparation

The chemical structures of the tested compounds (**1**, **4**, and **9a**–**i**) were built using MarvinSketch 22.11 software and optimized by Discovery Studio 2021 software using Dreiding-like forcefield (Hahn 1995). Gasteiger charges were applied to merge the non-polar hydrogens using AutoDock Tools 1.5.6.

### Protein preparation

The X-ray structure of both the wild-type B-Raf kinase domain (PDB ID: 1UWH) (Wan et al. 2004) and the mutated B-Raf kinase domain (PDB ID: 4FK3) (Tsai et al. 2008) were downloaded from the RCSB protein databank (Elkamhawy et al. 2024). Reference ligands, water molecules, and any additional chains were removed, keeping one kinase domain only. The hydrogen atoms and Asn/Gln/His flips were assigned using Molprobity. AD4 parameters and Gasteiger charges were assigned to the protein atoms using AutoDock Tools 1.5.6.

### Molecular docking protocol validation

Molecular docking calculations were performed using AutoDock4 using ten runs of generic algorism (GA) at the ATP binding site coordinates (B-Raf^WT^; x = 75.15, y = 44.83, z = 65.03 and B-Raf^V600E^; x = −1.99, y = 50.20, z = 20.15). The docking protocol was validated through running initial docking experiments (pre-docking) for the reference ligands and calculating the RMSD values. The molecular docking of the designed compounds (**9a**–**i**) and standard compounds (**1** and **4**) was conducted and the most stable conformers were identified.

### Molecular docking analysis

The docking poses of the tested compounds were visualized and analysed using Discovery Studio software 2021 to identify the binding interactions between the docked ligands and the key amino acid residues at the ATP binding site of both B-Raf isoforms.

## Results

### Chemistry

**Scheme 1:**
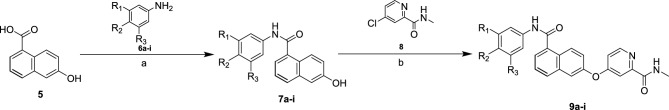
Reagents and conditions: **a)** T_3_P, DIPEA, THF, rt, 12 h; **b)** Cs_2_CO_3_, DMSO, 110 °C, 12 h

As shown in Scheme 1, the chemical synthetic procedure of the intermediate amide derivatives **7a**–**i** was carried out via coupling the commercially available 6-hydroxy-1-naphthoic acid (**5**) with different anilines (**6a**–**i**) in the presence of *N*,*N*-diisopropylethylamine (DIPEA) and propylphosphonic anhydride (T_3_P) in tetrahydrofuran solvent (THF) at room temperature (rt = 25 °C) for 12 h. The free phenolic OH group in compounds **7a**–**i** was then allowed to be reacted with 4-chloro-*N*-methylpicolinamide (**8**) in the presence of Cs_2_CO_3_ in DMSO at 110 °C for 12 h to afford the final target compounds **9a**–**i** (Table 1).

### Biological evaluation

#### In vitro kinase assay

To evaluate the new structural design of our compounds (**9a**–**i**), the biochemical activity was evaluated against Raf kinase enzymes (B-Raf^WT^, B-Raf^V600E^, and c-Raf) in one-point dose (10 μM) at duplicate mode using ATP concentration 10 μM. The results are presented in Table 2 and expressed in % inhibition. The results demonstrated that the majority of the compounds exhibited strong inhibition, exceeding 90%, across Raf kinases. In addition, the new structural mapping (Fig. 2) can suggest the potential binding into the ATP binding site in a competitive manner with ATP molecules. Notably, the newly introduced lipophilic groups at the distal phenyl ring (F, Cl, Me, OMe, OCHF_2_, and isopropyl) are expected to have a crucial role in binding interactions within the ATP binding site. Among the tested derivatives, compound **9e**, showed weak inhibition to the tested Raf kinases (% inhibition: 24.93–76.30%).

**Table 2:** % inhibition values of the tested compounds at 10 µM against B-Raf^WT^, B-Raf^V600E^ and c-Raf kinase enzymes

Compound	In vitro protein kinase inhibition values (%)		
	B-Raf^WT^	B-Raf^V600E^	c-Raf
**9a**	99.43	100.00	99.80
**9b**	98.41	98.40	99.74
**9c**	92.74	88.59	98.88
**9d**	97.41	98.16	99.57
**9e**	42.06	24.93	76.30
**9f**	74.69	75.80	96.48
**9g**	99.34	100.00	99.84
**9h**	99.61	100.00	99.77
**9i**	99.67	100.00	99.64

#### In vitro cytotoxic activity

The synthesized compounds (**9a**–**i**) were tested in the cellular level against the human melanoma cell line (A375) to determine the IC_50_ values using sorafenib as standard (Table 3). Most of tested compounds showed potent cytotoxic activities against melanoma cell lines compared to that of sorafenib. The results revealed that the tested compounds showed sufficient lipophilicity for the cellular conduction. Moreover, compounds **9a**–**d** and **9f** showed more cytotoxic activities against melanoma cell line (0.12–0.67 μM) compared to that of the standard (0.92 μM). In addition, compounds **9 g**–**i**, with dual substitutions at distal phenyl ring (diMe, Me/Br, and Cl/OMe, respectively), showed moderate cytotoxic activities compared to that of the standard. The mono-substitution at the distal phenyl ring of the designed compounds was found to be optimal for the inhibitory profile against melanoma cell line. Interestingly, compound **9d** with disubstituted phenyl ring (F/Me) showed potent cytotoxic activity (0.54 μM). It was expected that the small-sized F group would retain the cytotoxic activity as that of potent series with mono-substitution (**9a**–**c** and **9f**). On the other hand, compound **9e** (with a bulky and polar morpholine group) exhibited weak cytotoxic activity against melanoma cell line (A375) compared to that of sorafenib. Both results of the biochemical assays and cytotoxic evaluation of compound **9e** revealed that the introduction of bulky and/or polar groups at the distal phenyl ring is not favoured for the desired inhibitory profile.

**Table 3:** IC_50_ values (μM) of the tested compounds (**9a**–**i**) and standard (sorafenib) against human melanoma cell line (A375)

Compound	IC_50_ values (μM)	Compound	IC_50_ values (μM)
**9a**	0.12 ± 0.013	**9f**	0.44 ± 0.008
**9b**	0.17 ± 0.012	**9g**	1.42 ± 0.013
**9c**	0.67 ± 0.017	**9h**	1.67 ± 0.032
**9d**	0.54 ± 0.019	**9i**	1.75 ± 0.021
**9e**	4.46 ± 0.024	Sorafenib	0.92 ± 0.018

#### Dose-dependent kinase assay

A further dose-dependent assay was conducted for the most active compound (**9a**), evaluated through biochemical and cellular assays, to determine its IC_50_ values against the three Raf kinase isoforms, using sorafenib as the standard reference. Compound **9a** was tested in 10-dose IC_50_ mode with a threefold serial dilution starting at 20 μM at ATP concentration 10 μM (Table 4). The results revealed that compound **9a** exhibited potent IC_50_ values against the three isoforms in nanomolar level. Interestingly, it also exhibited expanded inhibition to the mutated B-Raf kinase (V600E) in contrast to sorafenib that did not show the same inhibitory profile over the mutated isoform.

**Table 4:** IC_50_ values of most active compound (**9a**) against B-Raf^WT^, B-Raf^V600E^ and c-Raf kinase enzymes (nM) using Sorafenib as standard

Compound	IC_50_ values (nM)		
	B-Raf^WT^	B-Raf^V600E^	c-Raf
Sorafenib (1)	22.01	38.22	6.13
**9a**	49.74	18.05	2.86

#### Kinase selectivity assay

To evaluate the selectivity profile of the designed compounds, compound **9a** (the most active compound among the tested series) was tested for its kinase inhibitory profile against a set of different kinases (related to the same signalling pathway) in one-point dose (10 μM) at duplicate mode using ATP concentration 10 μM. The results are summarized in Table 5 in % inhibition. It is not surprising for compound **9a** to show potent inhibition against FGFR1 (86.23%). This cross-inhibition was suggested because of the structural similarity between our newly designed compounds (**9a**–**i**) and sorafenib (reported to inhibit FGFR1) (Wilhelm et al. 2006). No significant inhibition was observed over the other tested kinases. From the cytotoxicity assay, it is suggested that the dual inhibition of pan-Raf kinases and FGFR1 has a promising biological profile in treating melanoma.

**Table 5:** Protein kinase screening of most active compound (**9a**) against MAPK signalling cascade-related kinases (% inhibition)

Kinase	% inhibition values of compound **9a** (%)
ALK	8.23
B-Raf^WT^	99.43
B-Raf^V600E^	100
c-Raf	99.8
ERK2/MAPK1	0
FGFR1	86.23
mTOR/FRAP1	0
ROS/ROS1	0
TRKB	17.27

### Cell cycle analysis of compound 9a on melanoma cell line A375

**Fig. 3:**
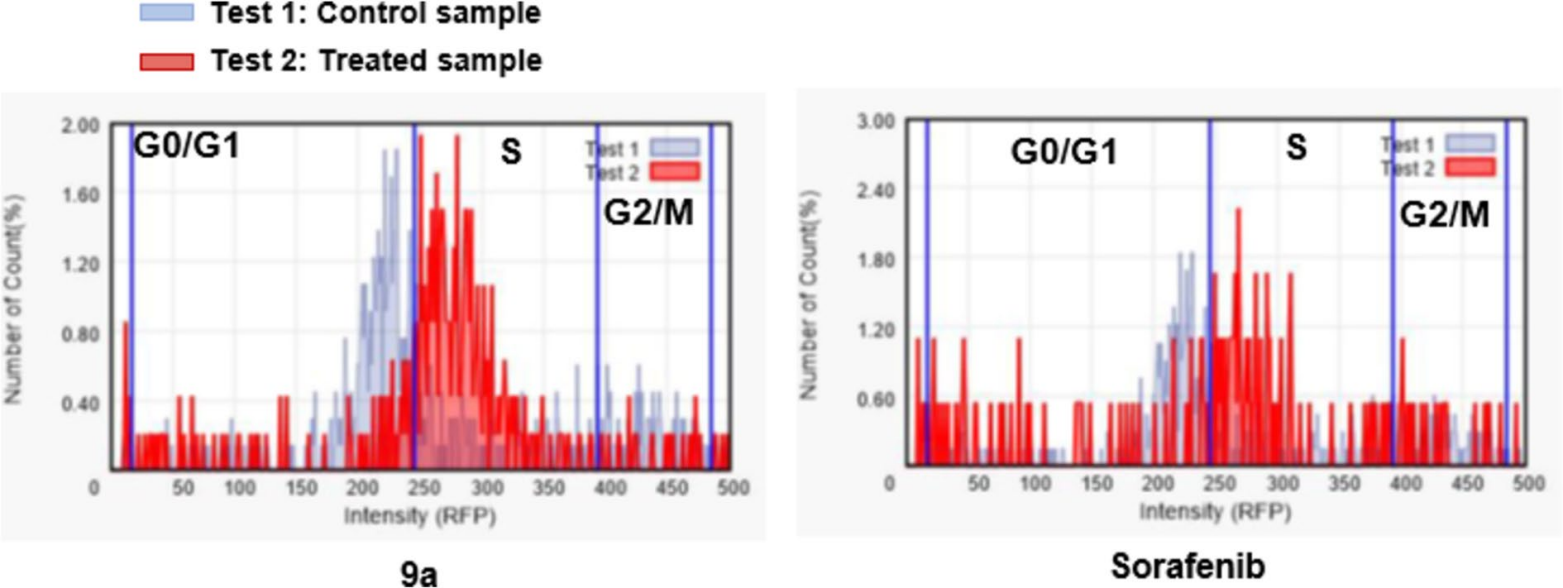
The effect of inhibitors on the phases of the cell cycle, compound **9a** and Sorafenib compared with control in A375 cells

The advancement of the cell cycle is accountable for regulating regular cell growth and multiplication. DNA damage may lead to either programmed cell death (apoptosis) or DNA repair. The cells' condition is assessed at distinct checkpoints, acting as control points to ensure accurate cell division. Key checkpoints in the cell cycle comprise G1 (restriction), S (metaphase), and G2/M, where the status of the cells is carefully examined (Soliman et al. 2019). The purpose of anticancer drugs is to interrupt cell division precisely at these checkpoints. Administering powerful cytotoxic agents, utilized as anticancer treatments, can pinpoint the phase in the cell cycle where apoptosis takes place. Accordingly, the most potent compound in our new series, **9a**, was selected for investigating its effects on the cell cycle profile and apoptosis. A375 cells were treated with compound **9a** at its IC_50_. The comparison data in Table 6 and Fig. 3 indicate that compound **9a** (Test 2) arrested the cell cycle of A375 cells at the *S* phase by 71.83% (Fig. 3). In addition, the cell population in G1 and G2/M phases decreased after treatment (Test 2) compared to negative control (Test 1). The comparison data showed the control sample has arrested the cell cycle at G0/G1 phases while **9a** treated sample has arrested it at *S* phase as indicated by higher number of counts (%) in these phases of both cell cycle studies. In contrast, sorafenib showed a different pattern, with a higher proportion of cells arrested in the G0/G1 phase (34.08%) and fewer in the S phase (49.72%), suggesting that sorafenib primarily disrupts cell growth earlier in the cell cycle. Additionally, sorafenib had a more pronounced effect in the G2/M phase (13.41%) compared to **9a** (6.45%), indicating its role in affecting cell division at the mitotic phase. Overall, compound the ability of compound **9a** to arrest the cell cycle on S phase suggests it is more effective at inhibiting melanoma cell proliferation, particularly at the DNA replication stage, while sorafenib targets earlier phases of the cell cycle, showing a different mechanism of action.

**Table 6:** The effect of compound **9a** and Sorafenib on the different phases of cell cycle

	Melanoma cells-A375					
	9a		Sorafenib		Control	
	Conc (cells/mL)	Percent	Conc (cells/mL)	Percent	Conc (cells/mL)	Percent
G0/G1 phase	1.16 × 10E4	18.92%	8.02 × 10E3	34.08%	4.95 × 10E4	58.27%
S phase	4.39 × 10E4	71.83%	1.18 × 10E4	49.72%	2.12 × 10E4	24.88%
G2/M phase	3.95 × 10E3	6.45%	3.16 × 10E3	13.41%	1.31 × 10E4	15.46%

### Apoptosis analysis of compound 9a on melanoma cell line A375

PE-conjugated Annexin V maintains its strong binding to phosphatidylserine (PS), serving as a sensitive marker for flow cytometric assessment of apoptotic cells. The staining with PE Annexin V precedes the loss of membrane integrity observed in the later phases of cell death, whether induced by apoptotic or necrotic processes. Therefore, PE Annexin V staining is commonly employed alongside a vital dye like propidium iodide (PI) or DAPI. This combination allows researchers to distinguish early apoptotic cells (DAPI negative, PE Annexin V positive) from viable cells with intact membranes, which exclude DAPI. In contrast, the membranes of dead or damaged cells become permeable to DAPI. Monitoring apoptosis over time reveals a progression of cells transitioning from PE Annexin V and DAPI negative (viable with no apparent apoptosis) to PE Annexin V positive and DAPI negative (indicative of early apoptosis), and ultimately to PE Annexin V and DAPI positive (representing end-stage apoptosis and cell death). The sequential movement of cells through these stages serves as an indicative pattern of apoptosis.

**Fig. 4:**
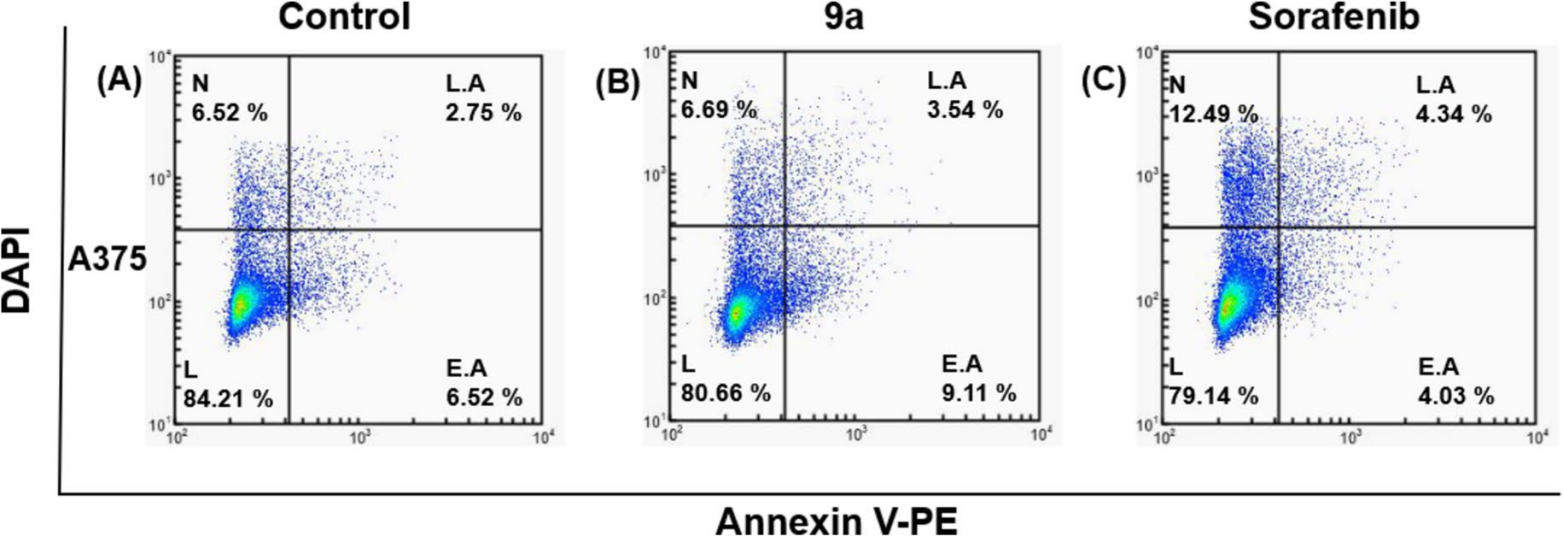
Dot plots showing apoptosis analysis of A375 cells (**A**, **B**, **C**) induced by compound **9a** along with **A** negative and **C** positive controls

**Fig. 5:**
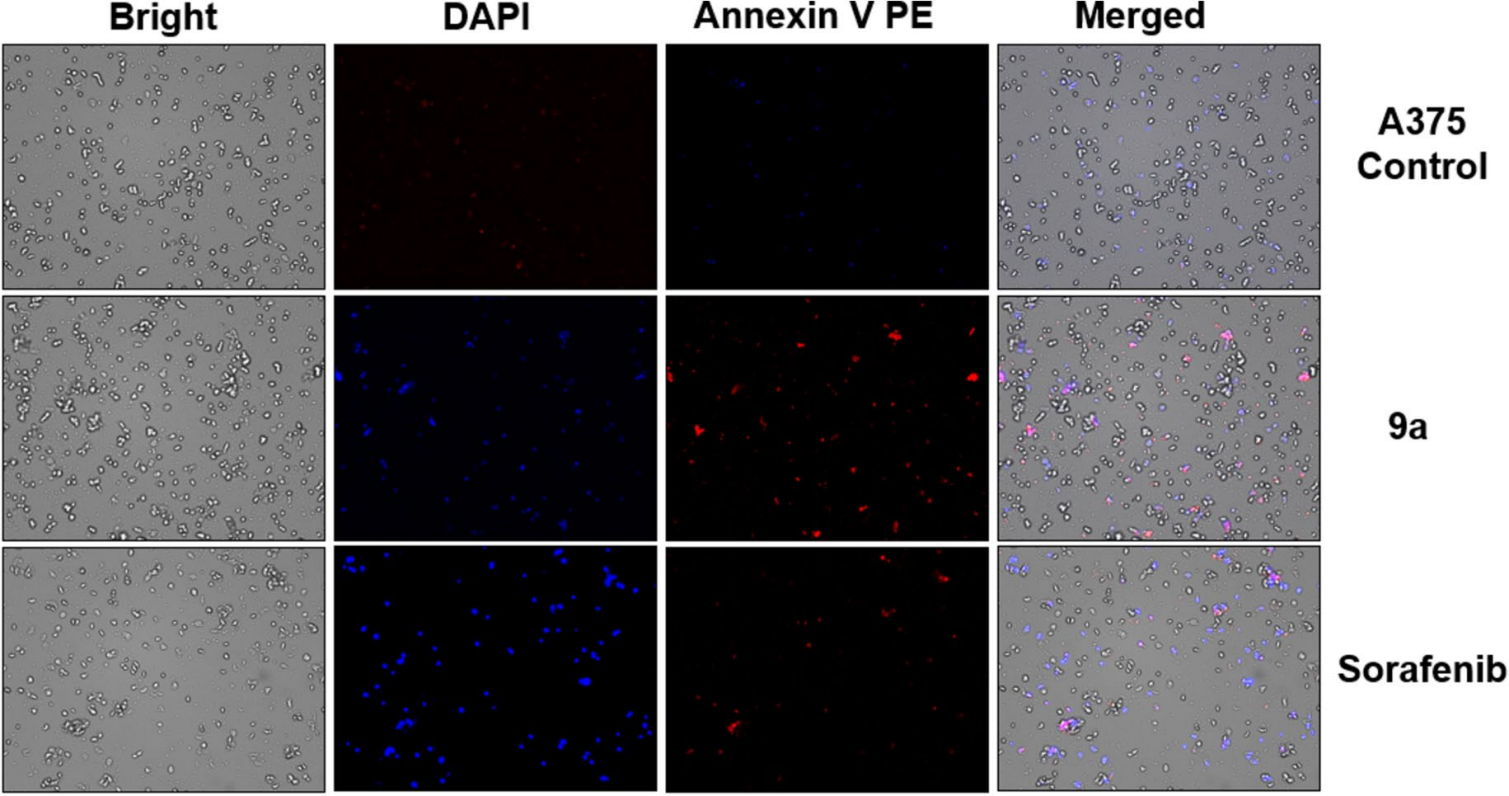
Intracellular fluorescence images of A375 cell line treated with **9a** for 24 h. Bright-field images, fluorescence images (DAPI:4′,6-diamidino-2-phenylindole, RF: red fluorescence), and merged images were assigned to the A375 Melanoma cancer cells with control (without any compound treatment), **9a** treated and Sorafenib treated, respectively showing apoptotic cells

For a more appropriate investigation of cell death, the compound-treated cells and control A375 cells were stained with PE-Annexin V and DAPI. The cellular fluorescence analysis was then carried out using ADAMII LS (Figs. 4 and 5). A kind of controlled cell death known as apoptosis may be identified with the use of Annexin V and the DAPI reagent. These two fluorophores are used in the dot plot and image data to identify early and late apoptotic cells. The findings of the dot plot analysis showed that a significant percentage of compound **9a**-treated A375 cells experienced early apoptosis (9.11%) (Fig. 4B) as compared to sorafenib (4.03%) (Fig. 4C), whereas some cells notably advanced to the late apoptosis phase. We discovered that most of the cancer cells in the control groups (about 84.21%) were alive (Figs. 4A, 5). Similarly, greater fluorescence of DAPI and Annexin V effectively separated compound **9a**-treated cells from control in the fluorescence images for A375 (Fig. 5) cells, which support the dot plot findings. As a result, when compound **9a** was applied at dosages close to its IC_50_ value, it damaged more A375 cells than the standard drug (sorafenib). The cell cycle and apoptotic studies' findings indicate that compound **9a** has exceptional anticancer capabilities that make it a promising candidate for chemotherapy.

### In silico molecular simulation

**Fig. 6:**
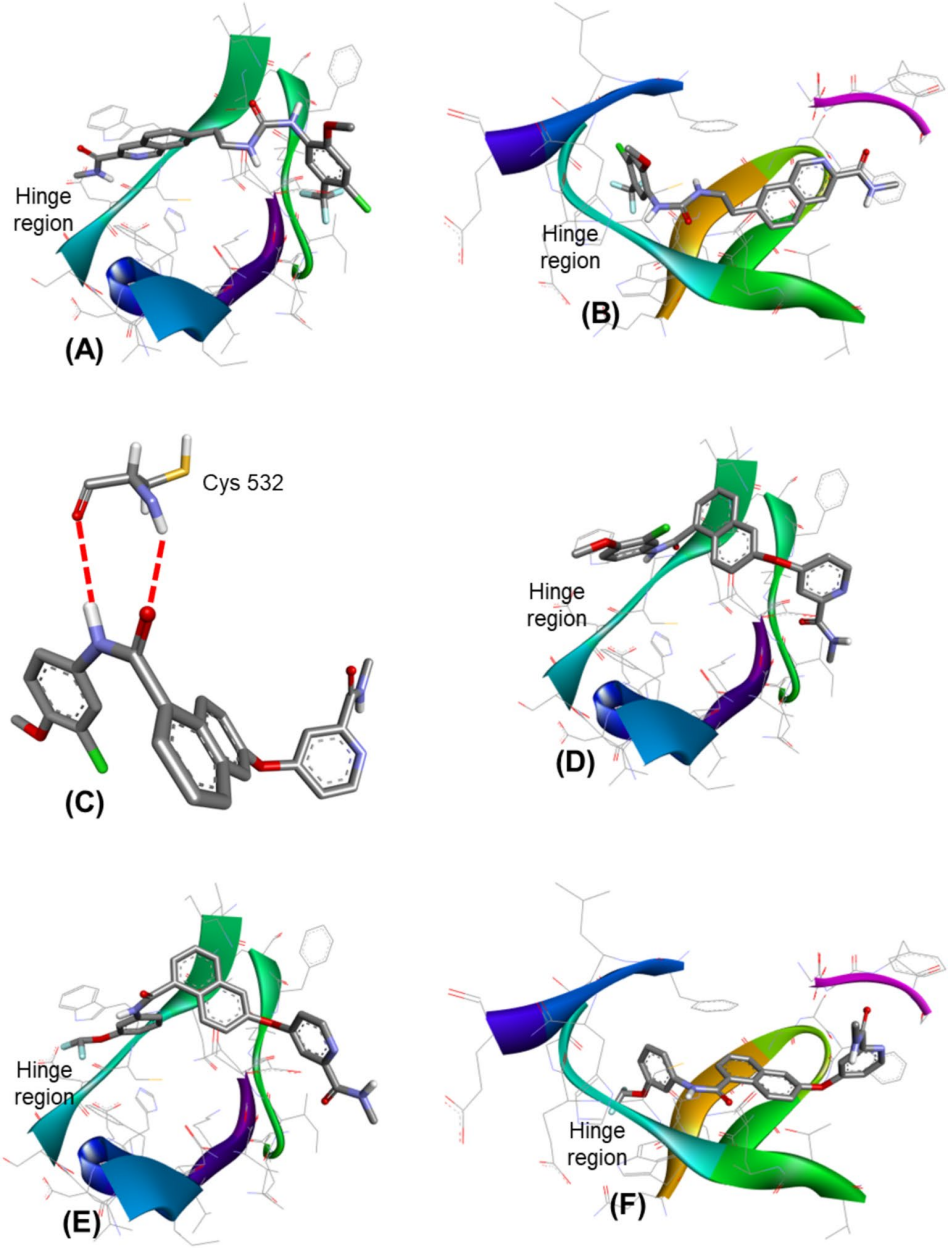
The in silico molecular docking simulation results. **A** the 3D interaction of compound **4** into B-Raf^WT^ active site showing terminal *N*-methyl amide group anchored towards the hinge region; **B** the 3D interaction of compound **4** into B-Raf^V600E^ showing the flipped conformation where the *N*-methyl amide group directed away from the hinge region; **C** the general structure of the newly designed compounds (Compound **9i** is shown here as a representative example) bound to the key amino acid residues at the hinge region (Cys532) showing strong H-bond interactions with the amide linker rather than the terminal *N*-methyl amide group. The bent conformation is identified in the designed series; **D** the 3D interaction of compound **9i** into B-Raf^WT^ active site showing the amide linker anchored towards the hinge region; **E** and** F** the 3D interaction of most active compound (**9a**) into B-Raf^WT^ and B-Raf^V600E^ active sites, respectively showing the substituted phenyl ring anchored towards hinge region where the difluoromethoxy group bound to the Cys532 amino acid residue with extended conformation

**Fig. 7:**
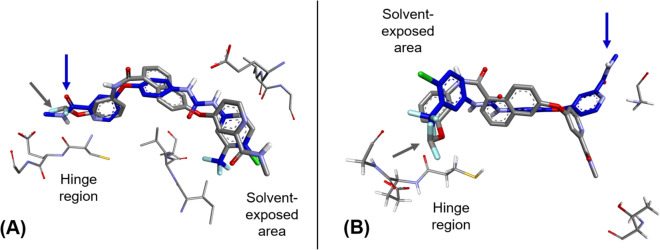
The 3D overlay representation of the generated conformations of both Sorafenib (**1**, blue backbone) and compound **9a** (grey backbone). **A** The generated conformations of both Sorafenib (**1**) and compound **9a** in B-Raf^WT^ ATP active site shows both motifs (*N*-methyl amide and difluoromethyl groups, respectively) anchored to the hinge region of the ATP binding site of wild-type isoform. **B** The generated conformations of both Sorafenib (**1**) and compound **9a** in B-Raf^V600E^ ATP active site shows flipped conformation of Sorafenib (**1**) where the conventional hinge binding motif (*N*-methyl amide) allocated away from the hinge region of the ATP binding site of mutated isoform exhibiting lower binding score (−8.19 kcal/mol) compared to that of compound **9a** (−9.32 kcal/mol)

To identify the possible binding interactions and the binding modes of the newly designed compounds, molecular docking simulation of the designed compounds (**9a**–**i**) was conducted into the ATP binding site of both wild-type B-Raf kinase domain (PDB ID: 1UWH) (Wan et al. 2004) and the mutated B-Raf kinase domain (PDB ID: 4FK3) (Tsai et al. 2008) using compounds **1** and **4** as standards (Table 7, Fig. 6 and Fig. 7). The most stable conformers of the tested compounds (**9a**–**i**) were selected among the generated conformers to explore, examine, and analyse the docking results and to discuss the possible binding interactions with the key amino acid residues within the ATP binding site. The docking results revealed that both standard compounds (**1** and **4**) exhibited the conserved binding pose into the ATP binding site of the wild-type B-Raf kinase domain (*N*-methyl amide group oriented at the hinge region; Cys532, Fig. 6A) with binding scores of −10.73 and −10.87 kcal/mol, respectively. However, these compounds (**1** and **4**) failed to show the same binding pose into the ATP binding site of the mutated B-Raf kinase domain (binding score = −8.19 and −8.34 kcal/mol, respectively). Where, they were flipped in such a way that the distal phenyl ring with non-polar substituents was anchored into the hinge area rather than the default binding motif, *N*-methyl amide group, and showed weak interactions with the key amino acid residues at that region (Figs. 6B and 7B). These docking results are aligned and matched with the limited inhibitory profile of sorafeni b over the mutated-B-Raf-based melanoma. On the other hand, the docking results of the newly designed compounds revealed that the new amide linker, between central naphthalene core-scaffold and the distal phenyl ring, favoured being oriented near the hinge region and bound with the key amino acid residues there (Cys532) across the wild-type and the mutated B-Raf kinase domains (Fig. 6C showed compound **9i** as a representative example of the newly designed derivatives) with binding score range of −9.73 −18.74 kcal/mol for B-Raf^WT^ and −8.11 −10.11 kcal/mol for B-Raf^V600E^ (Table 7). It was suggested that the 3D geometry of the amide linker of the newly designed derivatives, in contrast to the urea linker in both standard compounds (**1** and **4**), allows these compounds (**9a**–**i**) to fit, link, and bind with key amino acid residues at the hinge region in the ATP binding site. Moreover, the new binding mode of the tested compounds did not show the extended conformation, in contrast to that of compounds **1** and **4**. The binding of the amide linker into the hinge area led to the bent of the distal phenyl ring (with non-polar substituents) to be shifted to the upper surface of binding site (**9i**, Fig. 6D). It was surprising to find that the most active compound among the tested derivatives (**9a**) showed the extended conformation into the ATP binding site of both wild-type and the mutated B-Raf kinase domains. Additionally, it showed better binding scores than that of sorafenib into both the wild B-Raf and the mutated B-Raf kinase ATP active sites (**9a**, −10.49 and −9.32 kcal/mol; sorafenib, −10.73 and −8.19 kcal/mol in both wild B-Raf and V600E B-Raf, respectively). The terminal difluoromethoxy group showed a strong interaction with the hinge regions of kinase domain of both isoforms (Figs. 6E, F and 7).

**Table 7:** The binding scores (kcal/mol) of the synthesized compounds (**9a**–**i**) into the ATP binding site of kinase domain of both wild-type B-Raf (B-Raf^WT^) and mutated isoform (B-Raf^V600E^) using Sorafenib (**1**) and compound **4** as references

Compound	Binding score (kcal/mol)	
	B-Raf^WT^	B-Raf^V600E^
Sorafenib	−10.73	−8.19
**4**	−10.87	−8.34
**9a**	−10.49	−9.32
**9b**	−10.73	−8.14
**9c**	−10.34	−9.21
**9d**	−10.54	−8.23
**9e**	−9.77	−8.11
**9f**	−9.73	−8.13
**9g**	−10.01	−8.34
**9h**	−10.39	−8.79
**9i**	−10.74	−10.11

## Discussion

Vemurafenib and dabrafenib are FDA-approved drugs that target melanoma disease (Ali et al. 2022). However, acquired drug resistance is reported within six months from initiation of the drug therapy due to overexpression of other Raf isoforms (Rajakulendran et al. 2009; Shaw et al. 2014; Agianian and Gavathiotis 2018). Using sorafenib as a lead compound (Eisen et al. 2006), we developed a novel series (**9a**–**i**) designed to demonstrate an enhanced inhibitory profile across the Raf isoforms. We have conducted some structural modifications based on the chemical structures of sorafenib and compound **4** (Buchstaller et al. 2011). Naphthalene, a cytotoxic aromatic moiety that exhibits extensive biological activities, making it a valuable scaffold in drug discovery for various pathophysiological conditions, including anticancer, antimicrobial, and anti-inflammatory therapies (Makar et al. 2019) was suited as a central core scaffold in the designed compounds. The urea linker was replaced with an amide group, which is integral to many biomolecules and numerous clinically approved drugs, serving as a cornerstone in drug design (Kumari et al. 2020). Finally, the distal phenyl ring was substituted with different non-polar groups.

The in vitro kinase assay plays a critical role in evaluating the potency and selectivity of compounds, providing key insights into their potential as kinase inhibitors (Li et al. 2024). For this study, the in vitro kinase inhibition assays, including both one-point and ten-point evaluations, were conducted using the Kinase HotSpot service offered by Reaction Biology Co. (Malvern, PA, USA) to screen compounds **9a**–**i**. In particular, the assay was employed to assess the inhibitory potency of the newly synthesized compounds against Raf isoforms, including B-Raf^WT^, B-Raf^V600E^, and c-Raf as previously reported (Elkamhawy et al. 2016; Park et﻿ al. 2017). As shown in the results, it was suggested that the bulky and polar morpholine group introduced at position 4 in this derivative (**9e**) is not suitable at that position. A potential clash between the bulky morpholine ring and key amino acid residues in the binding pocket is anticipated, which could hinder the optimal fitting of compound **9e** into the ATP binding site. Consequently, this may reduce its competitive potential against ATP molecules at the binding site. In contrast, compound **9b**, featuring an isopropyl substitution at position 4, demonstrated potent inhibition against the tested Raf kinases. This suggests that the isopropyl group possesses an optimal size, making it a suitable modification for incorporation into the designed compounds without compromising their inhibition profile.

The drug discovery process is costly and requires comprehensive early-stage evaluation of drug candidates’ biological activity, toxicity, and mechanisms. Cell-based assays, with advantages like automation and predictability, are vital tools despite challenges (Michelini et al. 2010). Therefore, in vitro cytotoxic assay was performed against human melanoma cell line (A375). Most of the tested compounds showed significant inhibition across the Raf isoforms. However, compound **9e** (morpholine-containing compound) showed weak inhibition through the in vitro kinase assay and cytotoxic evaluation. The bulky and polar morpholine group is not tolerated in the designed series. Compound **9a** (difluoromethoxy group-containing compound) showed the most potent results across the in vitro assays. This newly introduced difluoromethoxy group at the distal phenyl ring (position 3) is proposed to enhance inhibitory activity by increasing affinity for the ATP binding site. While sorafenib contains a chemically similar trifluoromethyl group (CF_3_), it does not exhibit the same inhibitory profile against mutated B-Raf kinase as compound **9a**. The additional hydrogen atom in the difluoromethoxy group, combined with the oxygen atom spacer in compound **9a**, is believed to facilitate enhanced hydrogen-bond interactions (HBD and HBA, respectively) with key amino acid residues within the binding pocket of the mutated B-Raf kinase. Furthermore, compound **9a** demonstrated a broad inhibitory profile across all three Raf isoforms. These findings suggest that the structural modification significantly improved inhibitory activity compared to both sorafenib and compound **4** developed by the Buchstaller group. Additionally, the difluoromethoxy group is hypothesized to enhance the pharmacokinetic profile by shielding the molecule from metabolism at its most vulnerable sites (Me).

Molecular docking is a valuable tool for explaining the activity of biomolecules, defining molecular determinants for interaction with drug targets, and designing more efficient drug candidates (Gagic et al. 2019). In silico molecular docking simulations were performed on the tested compounds against both wild-type and mutated B-Raf kinase domains to explore their potential binding interactions, using sorafenib and compound **4** as reference standards. The newly designed compounds exhibited novel binding modes across both B-Raf isoforms, with the amide linker forming hydrogen bond interactions with the hinge region. This behavior contrasts with sorafenib and compound **4**, which formed hydrogen bonds with the hinge region of wild-type B-Raf via their terminal N-methyl amide groups (Buchstaller et al. 2011)*.* Compound **9a** also showed a unique binding interaction, where it was stabilized through an extended conformation into the ATP binding site of both wild-type and mutated B-Raf kinase domains. In particular, the terminal difluoromethoxy group at the distal phenyl ring showed strong interaction with the key amino acid residues at the hinge regions. This key binding mode can justify the potent activity of compound **9a** against the A375 melanoma cell line (IC_50_ = 0.12 μM) compared to that of sorafenib (IC_50_ = 0.92 μM). The docking results indicated that the hinge region preferentially interacts with the newly introduced terminal difluoromethoxy group in compound **9a**, stabilizing the extended conformation more effectively than the inner amide linker, which exhibited a less stable bent conformation. Furthermore, the difluoromethoxy group displayed essential chemical features for binding to key amino acid residues at the hinge region of both wild-type and mutated B-Raf kinase domains.

In conclusion, our findings demonstrate that the structural modifications of sorafenib, particularly the introduction of the difluoromethoxy group in compound **9a**, significantly enhanced its inhibitory activity across Raf isoforms, including mutated B-Raf and c-Raf. Compound **9a** exhibited superior potency in both kinase and cytotoxic assays, with a broad inhibitory profile and enhanced binding interactions at the ATP site, facilitated by the newly designed amide linker and terminal modifications. Meanwhile, future studies are suggested to explore a broader range of substituents to build on our findings, our results establish compound **9a** as a highly promising candidate for targeting Raf-driven malignancies, highlighting its superior potency and selectivity across Raf isoforms.

## Supplementary Information

Below is the link to the electronic supplementary material.

Supplementary file1 (DOCX 167 KB)

## Data Availability

The authors confirm that the data supporting the findings of this study are available within the article [and/or] its supplementary materials.
