# Supplementary material for: Discovery of novel naphthalene-based diarylamides as pan-Raf kinase inhibitors with promising anti-melanoma activity: rational design, synthesis, in vitro and in silico screening
Source: Arch Pharm Res. 2025 Feb 8;48(2):150–65. doi: 10.1007/s12272-025-01533-5 (PMC11861015; doi:10.1007/s12272-025-01533-5)
Supplement: Supplementary file 1 — Supplementary file1 (DOCX 167 KB) [file 12272_2025_1533_MOESM1_ESM.docx]

**Supplementary material**

1. **A375 Cell-based assay (Dose-response curves of compounds 9a-i & sorafenib)**


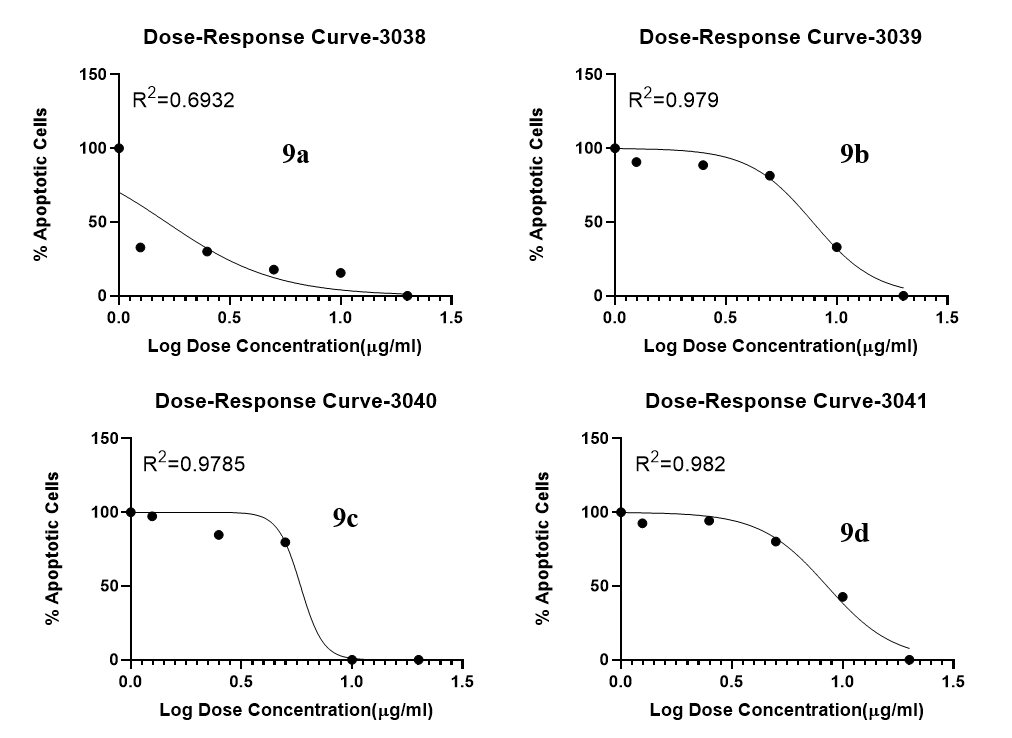


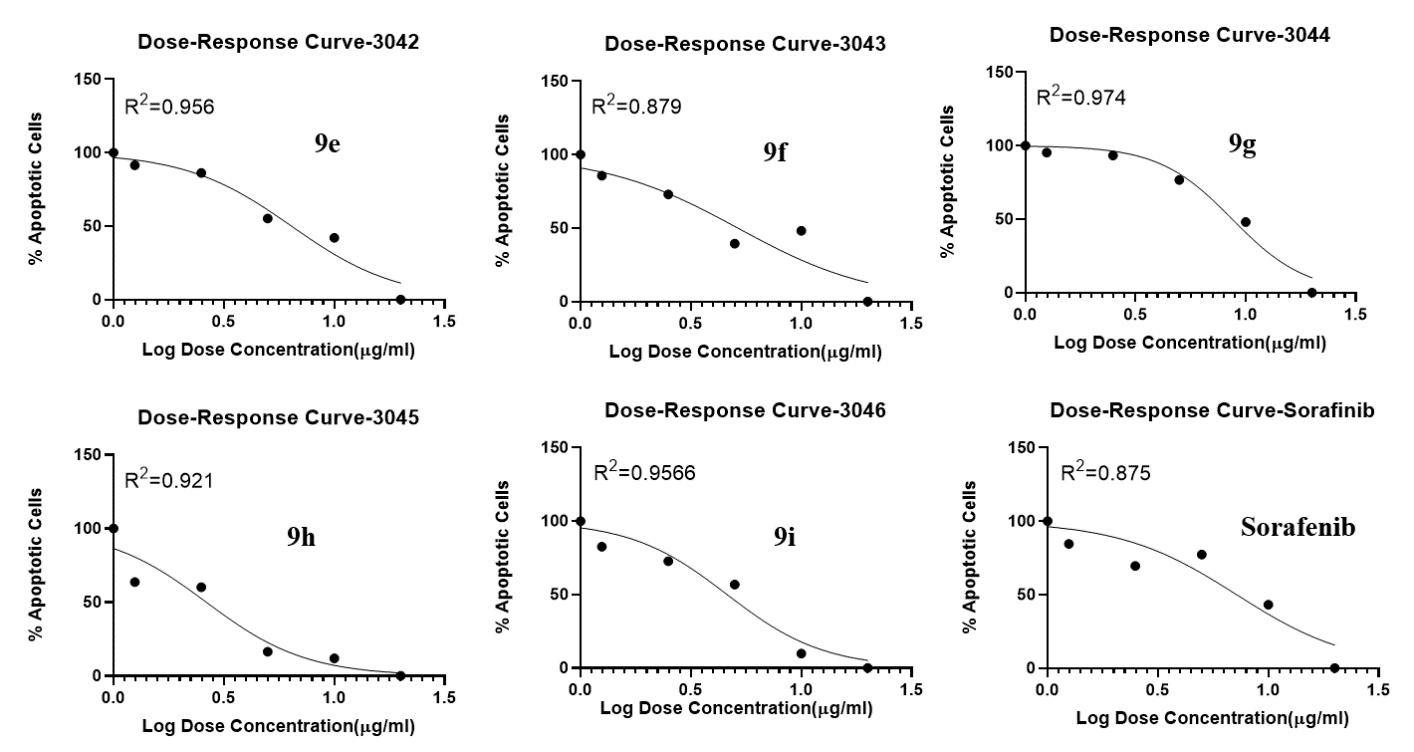


1. **Kinase-based assay (Dose-response curves of compound 9a over Raf isoforms)**
